# Differential mobility and local variation in infection attack rate

**DOI:** 10.1371/journal.pcbi.1006600

**Published:** 2019-01-22

**Authors:** David J. Haw, Derek A. T. Cummings, Justin Lessler, Henrik Salje, Jonathan M. Read, Steven Riley

**Affiliations:** 1 MRC Centre for Global Infectious Disease Analysis, Department of Infectious Disease Epidemiology, School of Public Health, Imperial College London, London, United Kingdom; 2 Department of Biology, University of Florida, Gainesville, Florida, United States of America; 3 Emerging Pathogens Institute, University of Florida, Gainesville, Florida, United States of America; 4 Department of Epidemiology, Johns Hopkins University, Baltimore, Maryland, United States of America; 5 Mathematical Modelling of Infectious Diseases Unit, Institut Pasteur, Paris, France; 6 CNRS, URA3012, Paris, France; 7 Center of Bioinformatics, Biostatistics and Integrative Biology, Institut Pasteur, Paris, France; 8 Centre for Health Informatics Computing and Statistics, Lancaster Medical School, Lancaster University, Lancaster, United Kingdom; UNITED KINGDOM

## Abstract

Infectious disease transmission is an inherently spatial process in which a host’s home location and their social mixing patterns are important, with the mixing of infectious individuals often different to that of susceptible individuals. Although incidence data for humans have traditionally been aggregated into low-resolution data sets, modern representative surveillance systems such as electronic hospital records generate high volume case data with precise home locations. Here, we use a gridded spatial transmission model of arbitrary resolution to investigate the theoretical relationship between population density, differential population movement and local variability in incidence. We show analytically that a uniform local attack rate is typically only possible for individual pixels in the grid if susceptible and infectious individuals move in the same way. Using a population in Guangdong, China, for which a robust quantitative description of movement is available (a travel kernel), and a natural history consistent with pandemic influenza; we show that local cumulative incidence is positively correlated with population density when susceptible individuals are more connected in space than infectious individuals. Conversely, under the less intuitively likely scenario, when infectious individuals are more connected, local cumulative incidence is negatively correlated with population density. The strength and direction of correlation changes sign for other kernel parameter values. We show that simulation models in which it is assumed implicitly that only infectious individuals move are assuming a slightly unusual specific correlation between population density and attack rate. However, we also show that this potential structural bias can be corrected by using the appropriate non-isotropic kernel that maps infectious-only code onto the isotropic dual-mobility kernel. These results describe a precise relationship between the spatio-social mixing of infectious and susceptible individuals and local variability in attack rates. More generally, these results suggest a genuine risk that mechanistic models of high-resolution attack rate data may reach spurious conclusions if the precise implications of spatial force-of-infection assumptions are not first fully characterized, prior to models being fit to data.

## Author summary

We know that some places have higher rates of infectious disease than others. At the moment, we usually only measure these differences for large towns and cities, though modern data allows us to track movement at much higher resolution. In this paper, we used a computer simulation of an epidemic to propose ways that rates of incidence in small local areas might be related to population density. We found that if infectious people are better connected than non-infectious people, perhaps because they receive visitors, then, on average, higher density areas would have lower rates of infection. If infectious people were less connected than non-infectious people then higher density areas would have higher rates of infection. As data get more accurate, this type of analysis will allow us to propose and test ways to optimize interventions such as the delivery of vaccines and antivirals during a pandemic.

## Introduction

The spatial heterogeneity of infectious disease incidence at large scales presents numerous intervention opportunities and challenges. Maps of malaria prevalence [1] have been used to target additional surveillance and to prioritize countries and geographical regions for additional intervention investment, resulting in substantial decreases in numbers of infections [2]. Over shorter timescales, spatial asynchrony in the northern hemisphere during the 2009 influenza pandemic likely led to variable effectiveness of vaccination when eventually deployed because of prior infections [3]. The epidemiological implications of substantial spatial heterogeneity in both incidence and transmission are topics of active research for most human pathogens [4].

These spatial heterogeneities must be influenced by two key human behaviours: where people choose to live and how they move. Because the home location of an individual is primarily used as the geographic location when cases are recorded, absolute spatial incidence is driven by population density: where more people live in a given unit area, there is greater potential for cases. Accurate high resolution estimates of population density [5, 6] and travel [7] have helped refine global absolute estimates of disease incidence and prevalence [8–11]. In order for a directly transmitted human pathogen to move through space, at least one person must travel away from home and meet another person. Even for vector borne pathogens such as malaria and Zika virus, typical distances traveled by the vector are much shorter than those traveled by human hosts. Human movement is captured by survey data on journeys to work [12], questionnaire-based surveys [13] and location logging of mobile devices [14–16].

Although spatial heterogeneity has been measured at larger scales (e.g. serological attack rates for influenza [17]), modern pathogen surveillance enables more finely resolved incidence data sets, with details such as precise geographical location captured with increasing frequency by modern digital and biological technology. For example, the full genome of a pathogen can be made available in almost real time directly from clinical samples taken in the community [18], and the home location of everyone attending a health care facility can be extracted from clinical episode data [19]. Because this level of geographical precision for high quality incidence data has not previously been available, both epidemiological and disease-dynamic studies of infectious disease have focused on predicting and explaining incidence patterns measured at larger spatial scales, often with all cases within an administrative unit reported together. Additional insights are likely being lost during this aggregation process.

Available evidence and intuition suggests that infectious and non-infectious individuals have different social interactions during an outbreak [20], with plausible scenarios in which either one or the other may be more connected in space. For example, susceptible individuals are more likely to travel more than are infectious individuals with mild symptoms [21]. However, family members and friends providing care for infectious individuals may often not behave in the same way as an average susceptible individual. Also, infectious individuals themselves may travel long distances away from transmission hotspots to seek medical care during outbreaks of highly pathogenic infections [22].

Disease dynamic models are often used to study infection incidence and are defined primarily by their force-of-infection (FOI) term: a precise mathematical specification of how the risk of infection experienced by a susceptible individual is driven by the number of currently infectious individuals and by their characteristics. For example, the ages of infectious and susceptible individuals must sometimes affect the risk of infection, as must the distance between their home addresses. Disease dynamic models that represent space [23] are now used routinely to understand large-scale spatial heterogeneity in incidence: to estimate the relative effectiveness of spatially heterogeneous interventions (given the observed incidence); to reveal underlying social mechanisms of transmissions; and, with increasing frequency, to forecast future spatial incidence patterns [24]. All transmission models that represent space include some kind of spatial kernel—a formal definition of the way in which individuals from different locations distribute their influence over the whole of geographical space.

However, there is substantial variability in the underlying FOI assumptions made in these models, which are often not discussed explicitly and have likely only rarely made material differences to model-based results aggregated at larger spatial scales. Nonetheless, we hypothesise that these different FOI assumptions represent important alternate hypotheses for the mechanisms of transmission and may lead to substantial structural biases in the predictions of attack rates at smaller spatial scales. Here, we propose a general theoretical framework for the study of infectious disease incidence at arbitrarily small spatial scales and, in particular, we look at the relative mobility of infectious individuals relative to susceptible individuals as a potential driver of heterogeneity in incidence.

## Results

Algebraic analyses show that differential spatial connectivity of susceptible and infectious individuals can lead to variability in local attack rates (S1 Protocol). Firstly, we showed that if susceptible and infectious individuals are assumed to be connected in the same way across all points in space, then local attack rates are uniform for any population density distribution or grid resolution. For lower resolution grids with large individual spatial elements, where the amplitude of connectivity of individuals outside their home pixel is small, the impact of differential connectivity between susceptible and infectious individuals is still negligible, even to the point that it is reasonable to assume that infectious individuals have no connectivity at all outside their home location. However, as the resolution of the grid increases and pixels become smaller, individuals have a substantial number of connections outside their home pixel. Under this scenario, it was no longer possible to prove analytically that differences in the connectedness of susceptible and infectious individuals would not lead to local variation in attack rates. These analytical results were not affected by the presence of age stratification in the transmission process, so long as the behavior and distribution of age groups was assumed to be uniform across space.

We established a baseline numerical scenario consistent with a 1918-like influenza pandemic by implementing the underlying transmission model (see Methods) as ordinary differential equations (ODEs). Using: a 1km by 1km gridded population density (55km by 33km to the east and north of Guangzhou, China); a spatial contact kernel estimated in the same population [25]; a basic reproductive number *R*_0_ = 1.8 [26] and recovery rate 1/2.6 days^−1^ [27]; we recovered a global uniform attack rate of *z* = 0.73, consistent with the homogeneous mixing model SIR model [28]. We also introduced age-stratified populations and transmission using parameters estimated in this population [13]. For this population, accurate high-resolution data on local age distributions were not available, therefore, we assumed that all pixels had populations with the same age distribution, even though the total number of individuals in a single pixel varied substantially. This addition of age effects in the transmission process did not introduce spatial variation but did reduced the uniform global attack rate to *z* = 0.43, consistent with analysis of the 2009 influenza pandemic [29]. We validated the precision of attack rates obtained from the ODEs using age- and space-stratified refinements [23] of the standard implicit equation relating attack rate (final size) *z* to *R*_0_: *z* = 1 − *e*^−*R*_0_*z*^ [28].

**Fig 1:**
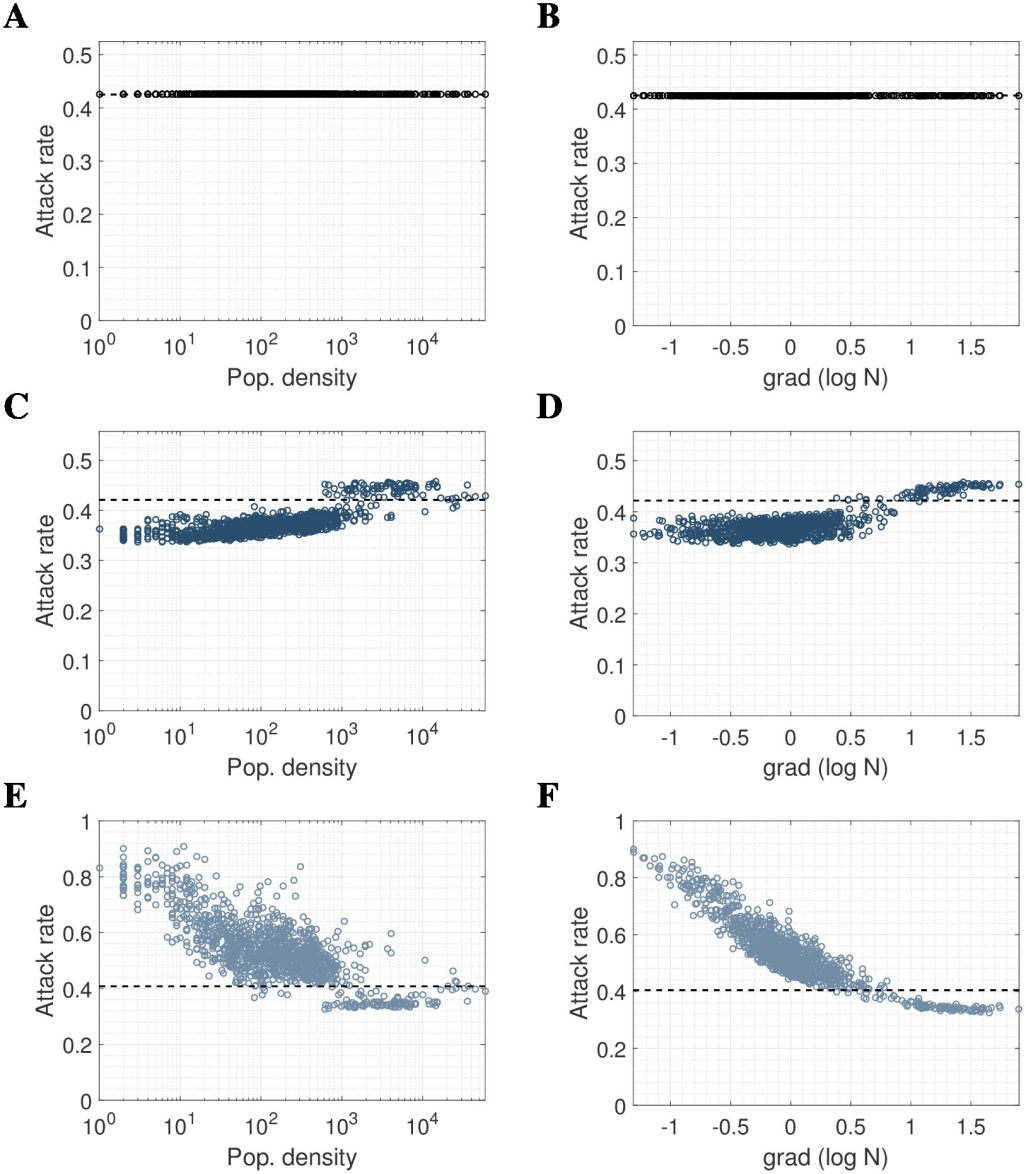
The relationship between force-of-infection (FOI) assumptions, local attack rates, population density and population density gradient, for a pandemic-influenza-like epidemic. The LHS shows the relationship between population density *N* (people/km^2^) and attack rate for **(A)** mobility independent of infection status (dual mobility), **(C)** mobility in non-infectious population only (S-mobility) and **(E)** mobility in infectious population only (I-mobility). The RHS shows the relationship between the gradient of log_10_N and attack rate for **(B)** dual mobility, **(D)** S-mobility and **(F)** I-mobility. We used a 33km by 55km grid of 1km by 1km pixels to the North-East of Guangzhou, with kernel parameters *α* = 0.52, *a* = 0.58, *p* = 2.72 and influenza natural history parameters *R*_0_ = 1.8, *γ* = 1/2.6. Population gradient was defined as the difference between the log population density of a pixel and the average log population density of the 8 surrounding pixels.

We hypothesized that both population density and the gradient of population density may influence small-scale attack rates in these models. Fig 1A and 1B show the uniform attack rate when mobility is independent of infection status (henceforth referred to as “dual mobility”) with four age classes, plotted against log of population density and gradient of log population density respectively (with log gradient defined as the average difference between the log of a location’s resident population and that of its 8 immediate neighbors).

**Fig 2:**
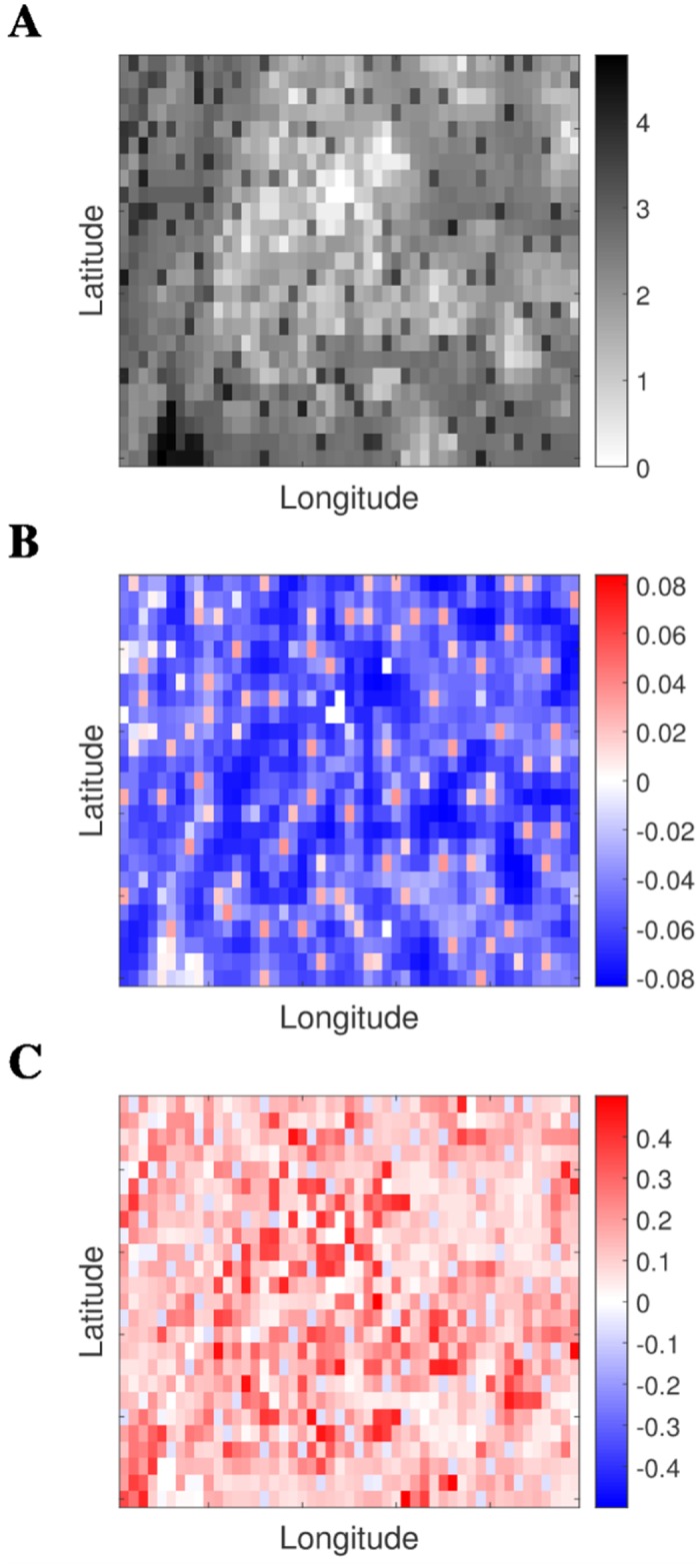
Spatial illustration of population density and non-uniform attack rates generated using different mobility assumptions. **(A)** Log_10_ population density (people/km^2^). **(B)** Difference between location-specific attack rates and global attack rate for S-mobility and **(C)** difference between location-specific attack rates and global attack rate for I-mobility. We change color scale between plots to better illustrate the emergent patterns. A total of 4 pixels are unpopulated and so attack rates are necessarily always zero in these locations.

When only non-infectious individuals were assumed to be mobile (S-mobility), location-specific attack rates were positively correlated with log population density, correlation coefficient c = 0.75 (Fig 1C). Attack rates varied between a minimum of 33.72% to a maximum of 45.76%, an absolute range of 12.04%. Location-specific attack rates were slightly less correlated with the log gradient of population density (correlation coefficient c = 0.73, Fig 1D). Locations with higher attack rates tended to be densely populated relative to neighboring locations (Fig 2A and 2B). Note that the term “S-mobility” includes mobility in the recovered population.

Conversely, when only infectious individuals were assumed to be mobile (I-mobility), pixel attack rates were negatively correlated with log population density (c = -0.7707, Fig 1E) and even more strongly negatively correlated with log density gradient (c = -0.8816, Fig 1F). Attack rates varied over a greater range than for susceptible-only mobility: from a minimum of 32.61% to a maximum of 90.73%, with an absolute range of 58.12%. High attack rate pixels tended to be sparsely populated relative to neighboring locations (Fig 2A and 2C). The reader is referred to the discussion for an evaluation of the applicability of this assumption to epidemic models.

**Fig 3:**
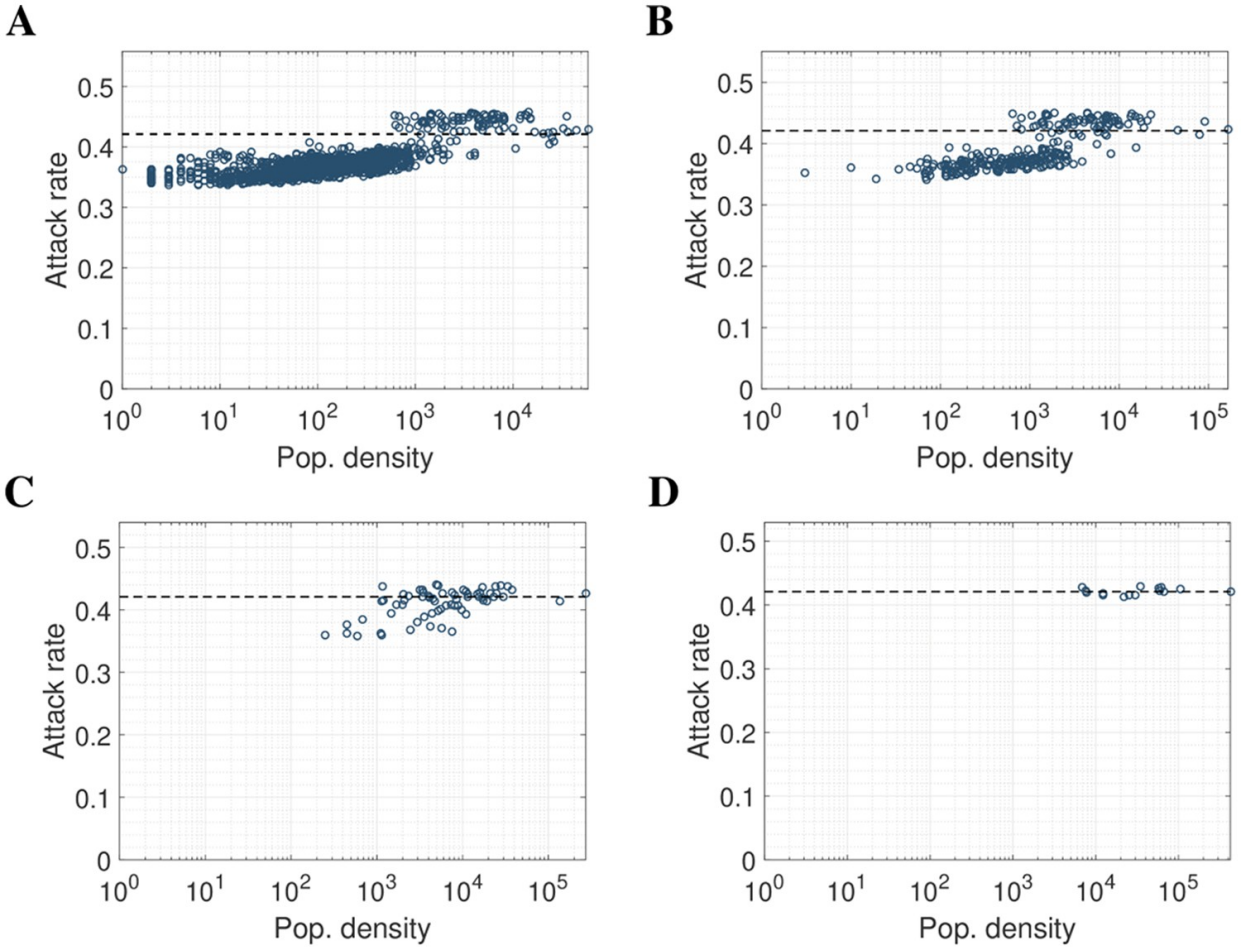
Aggregation of result using S-mobility. Plots show **(A)** initial result, aggregated into **(B)** 2km by 2km, **(C)** 4km by 4km, and **(D)** 8km by 8km pixels.

Measures of spatial variation are inherently dependent on the resolution of the model grid and even the strong variability outlined above would be missed by most surveillance systems. The absolute range of attack rates for the susceptible-only movement was reduced to 1.67% when aggregated to 8km by 8km pixels. Even though the effect of infectious-only movement was stronger than for susceptible-only mobility, it was rapidly hidden by the aggregation of pixels, with the absolute range dropping to 3.78% when aggregated to 8km by 8km pixels. Results of aggregation using S-mobility is shown in Fig 3, and the corresponding result using I-mobility is shown in S1 Fig.

**Fig 4:**
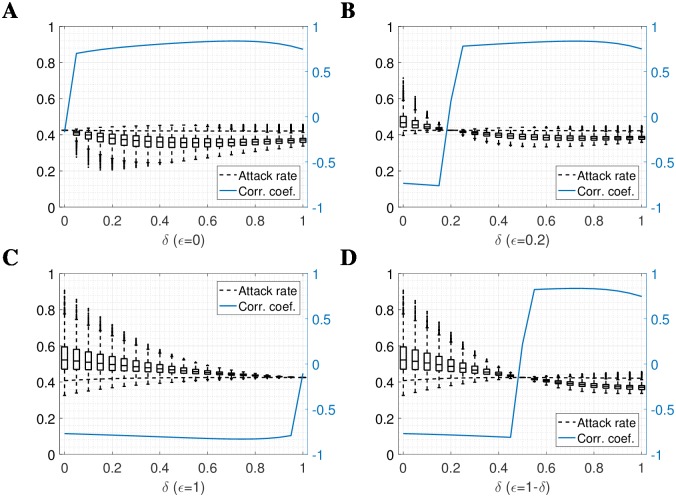
Limiting mobility of susceptible/recovered and immune agents according to parameters *δ* and *ϵ*. Mobility of the non-infective population is described by *δ* such that *δ* = 0 yields no mobility, *δ* = 1 yields mobility described by the kernel *K*, and transformation between these 2 extremes in linear. Similarly, *ϵ* describes the mobility of the infective population. Any values of *δ* = *ϵ* thus yield (reduced) dual mobility, and so attack rates are uniform in space. Plots show **(A)** infectious population immobile, non-infectious mobility ranging from *δ* = 0 to *δ* = 1, moving from dual mobility to S-mobility, **(B)** constant reduced mobility in the infectious population (*ϵ* = 0.2), possibly accounting for mobility in asymptomatic cases only, **(C)** full mobility in the infectious population, moving from I-mobility to dual mobility, and **(D)** *ϵ* = 1 − *δ*, illustrating the transition from I-mobility to S-mobility. Dashed lines show the global attack rate, and solid blue lines show correlation coefficient with log population density.

The direction of association between FOI assumptions and local attack rate was preserved and the amplitude remained substantial for intermediate scenarios in which both susceptible and infectious individuals were mobile but to differing degrees. If infectious individuals had any more contacts than susceptible individuals then attack rates were negatively correlated with population density, and vice versa (Fig 4). When infectious individuals reduced their travel by a factor of 0.5, the absolute range of attack rates was 5.38% and when susceptible individuals reduced their mixing by the same degree (with infectious agents fully mobile), the absolute range was 12.89%.

**Fig 5:**
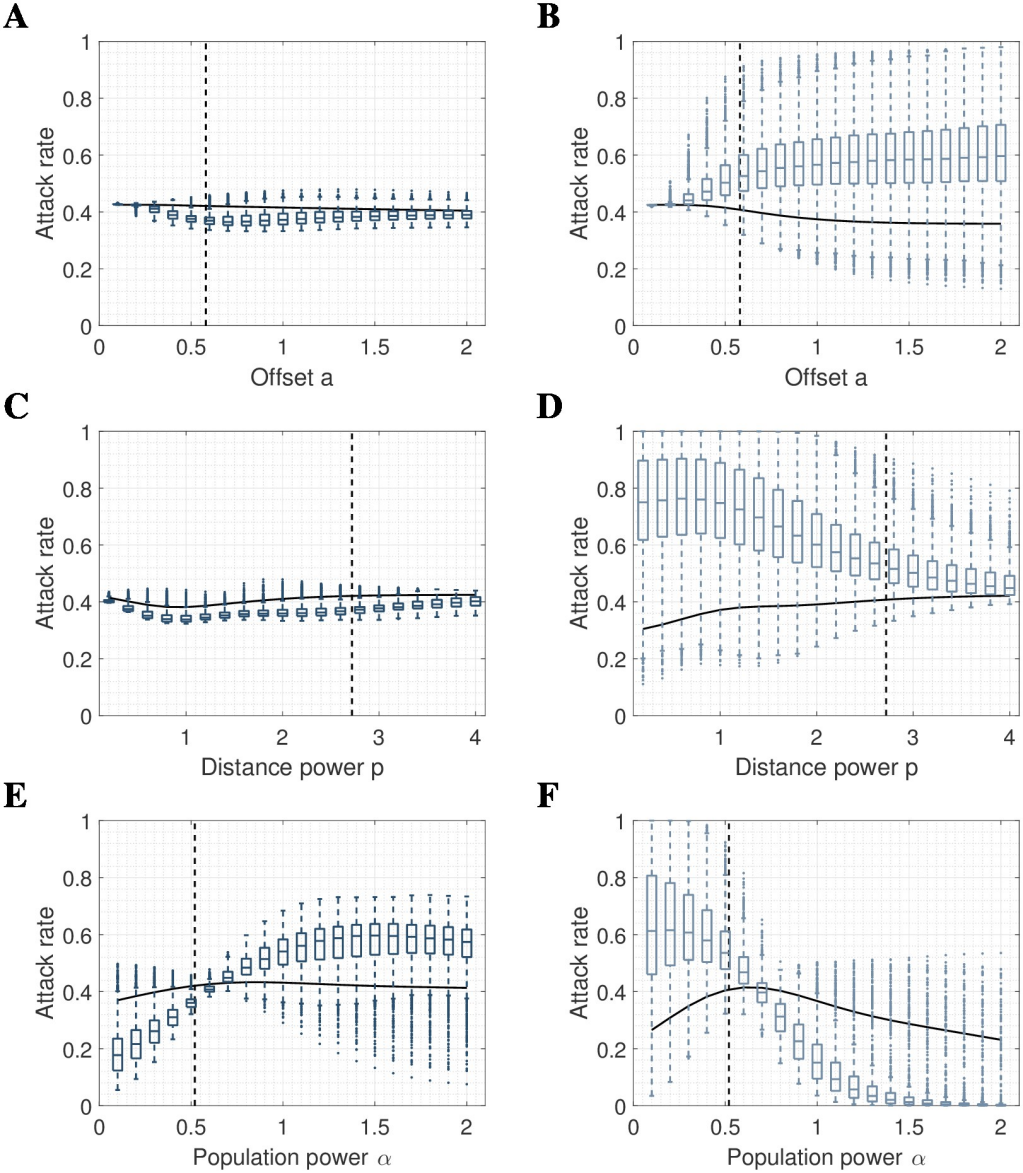
Sensitivity analysis. Distribution of local attack rates with respect to **(A)** offset *a* using S-mobility. **(B)** offset *a* using I-mobility, **(C)** distance power *p* using S-mobility, **(D)** distance power *p* using I-mobility, **(E)** population power *α* using S-mobility, and **(F)** population power *α* using I-mobility. Box plots show standard percentiles and outliers, solid lines show global attack rate, and dashed lines show parameter values used in the main result. When fixed, all parameters are as in main result, i.e. *a* = 0.58, *p* = 2.72, *α* = 0.52. Dual mobility are omitted as they are flat with variance *σ*^2^ = 0. Empty pixels yield attack rate zero and are omitted from calculations.

The underlying mobility choice kernel *K* was defined by the relative probability of making a contact in a population at a distance *r* and of population size *N*. It was parameterized by an offset distance *a*, a distance power *p* and destination population power *α*; *K* = *N*^*α*^(1 + *r*/*a*)^−*p*^, with values obtained by fitting to data from this population [25]. Qualitatively, our conclusions about the impact of differential contact rates by susceptible individuals were not sensitive to values for the offset distance *a* nor the distance power *p* (Fig 5A–5D). However, they were sensitive to values of the destination power *α* for which we have used the best fit value of 0.53 (for results up to this point) (Fig 5E&5F). Intriguingly, with the often-assumed default value *α* = 1, the correlation between local attack rates and population density or gradient have the opposite sign (S2 and S3 Figs). Moreover, *α* = 1 induces weaker correlations with local population gradient. It is therefore essential to provide an accurate estimate for *α*, which does not require infection-related data, before attempting to infer infection-dependent mobility.

**Fig 6:**
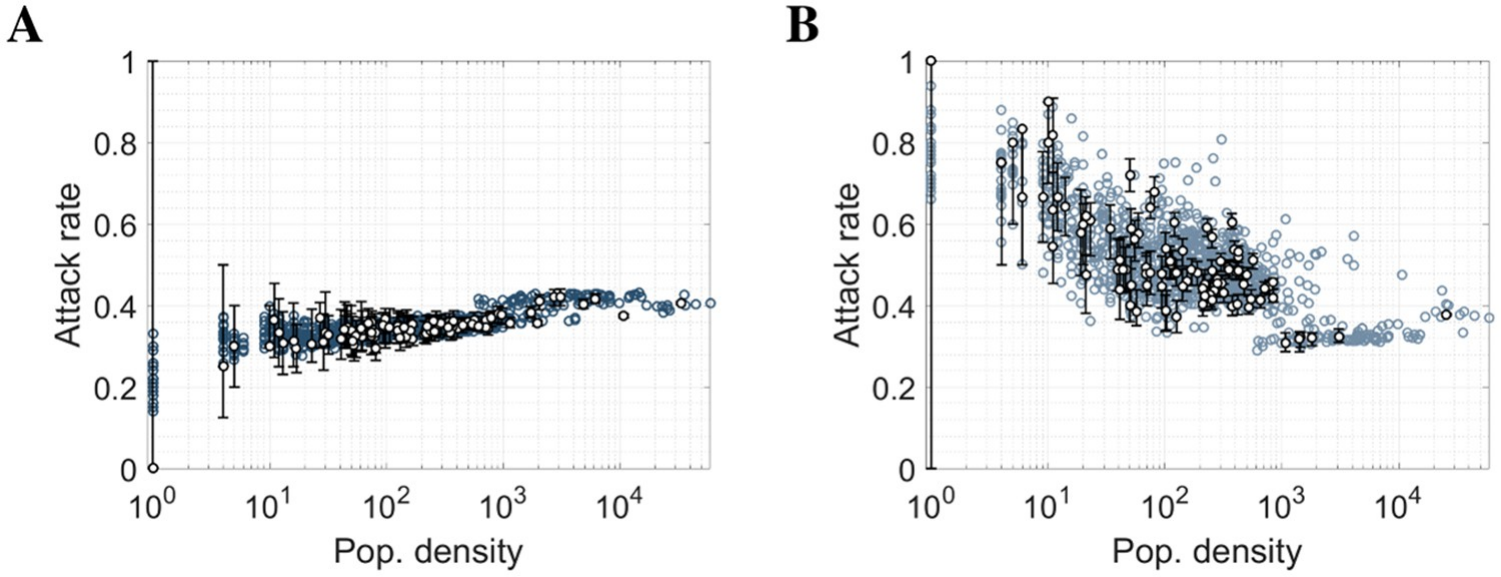
Mean attack rate over 100 iterations of stochastic equivalent of main result. We use **(A)** S-mobility and **(B)** I-mobility. 25-, 50- and 75-percentiles are shown for a sample of 100 locations.

Stochastic solutions to the meta-population models suggest that attack rate variation driven by asymmetric mobility would not be dominated by demographic stochasticity (Fig 6). Variation in attack rate for the extreme cases of S- and I-mobility was dominated by stochastic effects only in sparsely populated areas. For pixels with the smallest population, the amplitude of variation expected to arise from asymmetric mobility is similar to that which may arise by chance due to stochastic effects. However, the expected amplitude of stochastic variation diminishes as population density increases, and variation in attack rate due to mobility assumption becomes apparent (S4 Fig). For example, using susceptible-only mobility for 1km by 1km pixels with populations between 1 and 85,163, the standard deviation in attack rate due to stochasticity is 9.45% while the standard deviation of expected attack rates due to asymmetric mobility is 2.61%.

These results are robust to our choice of illustrative population density and to alternate natural history parameters. The same effects are observed when using population density of Puerto Rico with influenza natural history parameters (S5 Fig) and with parameters that approximate vector-borne transmission, such as those of Zika or Chikungunya (S6 Fig). Summary statistics for these and all other deterministic model variants we have presented in this study are shown in S1 Table.

## Discussion

We have shown that, under the assumption that an individual’s total contact is independent of home location and where they travel, substantial heterogeneity in local attack rates could arise if mobility is dependent on infection status. Moreover, the direction of the relationship between attack rate and population density is dependent on the contribution of population density to the relative attractiveness of a location. For the estimate of that scaling for our sample population (*α* = 0.52), and when susceptible individuals are more mobile than infectious individuals, attack rates are positively correlated with population density. Conversely, when using the often implicit assumption that the kernel is directly proportional to population density (*α* = 1), this correlation is negative.

Though increased mobility in infectious agents may seem less likely than reduced mobility, there do exist potential scenarios where this may be the case in both human and animal systems. For example, humans may travel to access health care in the case of severe symptom onset as has been the case anecdotally during the 2003/4 SARS outbreak and the 2013/14 Ebola outbreak. Also infectious opiate users in the USA may be more mobile than less infectious opiate users [30]. I-mobility may in fact be more relevant in the epidemiology of non-human infections, for example increased mobility in rabid dogs [31] and Gypsy moth caterpillars infected with baculovirus forfeit [32].

Our study has a number of limitations. We have not considered spatial variation in the age distribution of people, because these data were not available for our study population. Variability in local attack rates will very likely also be driven in non-trivial ways by spatial correlation in the proportion of the population in different age classes. This may be of particular significance in larger Chinese cities such as Guangzhou, in which urban areas are home to relatively few children and many rural locations have few working-age adults. There is also scope for the inclusion of an urban/rural distinction in the parametrization of the travel kernel [25], and the simulation of multiple years of transmission, which would extend the applicability of our results beyond pandemic scenarios for influenza and other emergent pathogens. The refinement of this framework to include the above phenomena is a priority for future work and we would expect differential movement patterns with age and population to impact our findings.

Though this study was limited to a standard SIR model, we would not expect the inclusion of a latent period, waning, or natural births and deaths to show make substantial differences to these findings. The primary results can be obtained using renewal equations which are only dependent on the probability of one individual escaping infection.

Our sensitivity analysis with respect to kernel population power *α* provides some insight into the underlying mechanisms that give rise to the observed correlations between attack rate and population density under different mobility assumptions. For example, consider the special case where only infectious people are mobile and *α* tends to large values, making mobility dependent only on population density of location, and not on geographical distance. Under this scenario, high density pixels will draw in more and more infectious people and therefore generate higher attack rates. Conversely, if *α* = 0, then mobility is dependent only on distance. Under this scenario, we can think of the infectious populations spilling out of their home locations into neighboring ones. Thus, any sparsely populated location that is adjacent to a densely populated location will see an influx of infectious individuals resulting in a greater *proportion* infectious in that location, and therefore a stronger FOI and subsequent attack rate. A schematic for the latter case is given in S7 Fig.

These results illustrate the potential knock-on effects of little or no dependence between transmissibility and population density: that infectious people from more densely populated areas go to nearby sparsely populated areas and in some sense “seek out” people in those areas to infect so they can reach their quota (I-mobility). Within the realm of parameters that are supported by studies of human movement and infectious processes, the behaviors implied by the models we presented here seem valid.

Individual-based models have a number of advantages over other approaches. They can be coded in a generic way and adapted rapidly to different pathogen systems and specific scientific or policy questions. Even though they are often more substantially computationally burdensome than comparable meta-population approaches, they will likely be used with increasing frequency to address questions related to local attack rates. We have shown that mobility assumptions have implications for the interpretation of attack-rates derived from individual-based models, some of which assume implicitly that the spread of infection is driven by the movement of individuals. We have shown that, whichever mobility assumption is made in a given model, it is possible to modify this assumption by replacing isotropic *K* by a convoluted kernel *L* that accounts for the change in mobility assumption (and so *L* may not be a stochastic matrix and hence functions as a non-isotropic kernel). In particular, the low-prevalence assumption makes this transformation achievable with minimal modification to existing computer programs. Therefore, developers of individual-based models may wish to consider alternate connectivity matrices for their simulations so as to explicitly reflect different spatial assumptions about the force of infection.

We have also shown that the implications of typical assumptions that are made in spatially explicit FOI terms, including approximations to this crucial normalization, are non-trivial at small spatial scales. Such assumptions are, however, often not addressed explicitly and so may contribute unknowingly to results. We hope to offer clarity in the interpretation of FOI in spatial models, and to have provided a comprehensive framework from which we can gain a deeper understanding of the role of spatial mobility in disease transmission dynamics as infectious disease incidence data become available at higher and higher spatial resolution.

## Methods

### Spatial kernels

Data taken from populations we study here show that total contacts made per day, and contact durations, do correlate with population density (*p* < 0.001, [13]), but that the strength of the relationship is weak. This is in part due to working-age adults dominating the population of urban areas, but also to the phenomenon of urban isolation [33]. When investigating only the effect of mobility assumption in force of infection, our main results made the baseline assumption that total contact and duration of contact is independent of home location.

The way in which these contacts are distributed in space does, however, depend on distance and population density, and is described via a spatial kernel *K*. In matrix notation, *K*_*ij*_ is defined as the proportion of time spent by an agent from location *i* in location *j*. The assumption of uniformity of total contact therefore means that the rows of *K* sum to unity. Our model employs the offset gravity kernel, defined as follows:

$$\begin{array}{c} K_{ij}\propto \frac{N_{i}N_{j}^{\alpha }}{1+{\left(r_{ij}/a\right)}^{p}} \end{array}$$ (1)

with baseline parameters of *a* = 0.58, *p* = 2.72, *α* = 0.52, where *r*_*ij*_ denotes the geodesic distance between the center-points of pixels *i* and *j*. Of the kernel structures studied in [25], offset gravity is shown to best represent contact data. Imposing the constraint that *K* is stochastic renders redundant the factor *N*_*i*_ in the numerator (owing to row-normalization).

### Population density map

We used rectangular excerpts from the Landscan dataset [34] with the lower left corner of the rectangle located on the center of the city of Guangzhou, China. The rectangle is 55km from east to west and 33km from north to south, and a 4km boundary area was excluded after simulation.

The boundary area was chosen according to the following rationale: when population density data for large suburban areas is truncated for the purpose of simulation, it is equivalent to imposing empty space outside of the boundary, and this modification may effect the attack rates calculated in pixels close to that boundary. We ran simulations on a large area of 1km by 1km pixels, and on smaller areas contained within this larger area. We found that attack rates agree on all pixels on the interior of the smaller area once a 4km perimeter is removed.

### Force-of-infection

Let *A* denote the S-mobility kernel and *B* the I-mobility kernel. Then the age-independent generalized FOI equation is given by:

$$\begin{array}{ccc} \lambda_{i} & = & \beta \sum_{j}A_{ij}\frac{\sum_{k}B_{jk}^{T}I_{k}}{\sum_{l}[A_{jl}^{T}\left(N_{l}-I_{l}\right)+B_{jl}^{T}I_{l}]}. \end{array}$$ (2)

For reduced mobility, movement of the non-infectious population is governed by a parameter *δ* such that *A* = (1 − *δ*)*E* + *δK*, where *E* is an identity matrix representing absence of spatial mobility. Similarly, we describe mobility of infective individuals by *ϵ* such that *B* = (1 − *ϵ*)*E* + *ϵK*. S-mobility thus corresponds to *δ* = 1, *ϵ* = 0 and I-mobility to *δ* = 0, *ϵ* = 1.

If *K* is the *n* × *n* spatial kernel, indexed by *i*, *j*, *k*, *l*, and *C* the 4 × 4 age-mixing matrix, indexed by *a*, *b*, *c*, *d*, then the age-explicit dual-mobility equation is given by:

$$\begin{array}{ccc} \lambda_{\left(a,i\right)}^{D} & = & \beta \sum_{b,j}K_{ij}\delta_{ab}\frac{\sum_{c,k}K_{jk}^{T}C_{bc}I_{\left(c,k\right)}}{\sum_{d,l}K_{jl}^{T}N_{\left(d,l\right)}} \end{array}$$ (3)

This can be combined with Eq (2) to give the age-dependent system with reduced mobility.

In all simulations presented in this study, we use the pointwise product of the matrices defining number of contacts and duration of contact between age groups 0–4, 5–19, 20–64 and 65+ derived in [13]. These age-mixing matrices were constructed from contact surveys conducted in the region of Guangzhou used in our results.

### Model solutions

We define the gridded transmission model as ordinary differential equations. However, we also implement a stochastic compartmental version of the model and we calculate attack rates using recursive equations.

We used a standard SIR model with ${\dot{S}}_{i}=-S_{i}\lambda_{i},\;{\dot{I}}_{i}=S_{i}\lambda_{i}-\gamma I_{i},\;{\dot{R}}_{i}=\gamma I_{i}$. ODE models were seeded proportional to population density (*σ* = 10^−8^ × **N**/∑_*i*_*N*_*i*_), and agreed with final size calculations (which assume infinitesimal seeding). Integration of ODEs with full FOI in the S- and I-mobility case, i.e. with *I*_*l*_(*t*) in denominators, showed low-prevalence approximations to be good. For example, in the main S-mobility result, the mean difference in pixel attack rates between the full FOI and low prevalence approximation was 6.22 × 10^−4^ with maximum difference 3.3 × 10^−3^ occurring in a pixel with population 726. Therefore, numerical solutions for all figures were obtained using the low prevalence approximation (c.f. S1 Protocol). A selection of smaller examples agreed when checked using the full FOI.

The stochastic compartmental variant of our model selected the number of agents to infect from binomial distribution with parameters *S*_(*a*,*i*)_ and 1 − exp(−λ_(*a*,*i*)_). This method requires specification of a time-step, and we found Δ*t* = 1/6 days to be sufficiently small (results did not change when Δ*t* was doubled, and results were consistent with the corresponding deterministic model).

## Supporting information

S1 Table Summary statistics for different model parameters, populations and mobility assumptions. Results for different grid sizes involve aggregation of result obtained at 1km by 1km resolution. In all cases, empty pixels are omitted from calculations. It is therefore possible to obtain a smaller minimum value of attack rate after aggregation.
(PDF)

S1 Fig Aggregation of result using I-mobility. Plots show **(A)** initial result, aggregated into **(B)** 2km by 2km, **(C)** 4km by 4km, and **(D)** 8km by 8km pixels.
(TIFF)

S2 Fig Sensitivity analysis: Correlation coefficient of attack rate with population density for different values of kernel parameters. We vary**(A)** *α* with *a* = 0.58 and *p* = 2.72 fixed, comparing S-mobility with I-mobility **(B)** *a* and *α*, using S-mobility with *p* = 2.72 fixed, **(C)** *a* and *α*, using I-mobility with *p* = 2.72 fixed, **(D)** *p* and *α*, using S-mobility with *a* = 0.58 fixed, and **(E)** *p* and *α*, using I-mobility with *a* = 0.58 fixed. All fixed parameter values are those used in main result.
(TIF)

S3 Fig Repeating our main result with *α* = 1. We use **(A)** S-mobility, with attack rates plotted against population density, **(B)** S-mobility/gradient, **(C)** I-mobility/density, and **(D)** I-mobility/gradient. Other parameters remain as in main result, i.e. *a* = 0.58, *p* = 2.72.
(TIF)

S4 Fig Ratio *R* of location-specific standard deviation over 100 iterations of stochastic model to standard deviation of corresponding deterministic model result over all pixels. We use **(A)** S-mobility and **(B)** I-mobility. All parameters as in main result, i.e. *a* = 0.58, *p* = 2.72, *α* = 0.52.
(TIF)

S5 Fig Simulated attack rates using population density of North-East Puerto-Rico: Influenza. We use a 60km by 60km grid of 1km by 1km pixels, and influenza-like natural history parameters *R*_0_ = 1.8, *γ* = 1/2.6, with **(A)** S-mobility plotted against population density, **(B)** S-mobility plotted against log population gradient, **(C)** I-mobility/density, and **(D)** I-mobility/gradient. Kernel parameters as in main result, i.e. *a* = 0.58, *p* = 2.72, *α* = 0.52.
(TIF)

S6 Fig Simulated attack rates using population density of North-East Puerto-Rico: Zika. We use a 60km by 60km grid of 1km by 1km pixels, and natural history parameters *R*_0_ = 4, *γ* = 1/10 approximating vector-borne transmission (e.g. Zika, Chikungunya), with **(A)** S-mobility plotted against population density, **(B)** S-mobility plotted against log population gradient, **(C)** I-mobility/density, and (D) I-mobility/gradient. Kernel parameters as in main result, i.e. *a* = 0.58, *p* = 2.72, *α* = 0.52.
(TIF)

S7 Fig Schematic illustration of the process by which the observed trends arise. As an example, assume infectious-only mobility and let location *x* be locally densely populated, with disease prevalence initially proportional to population density (initial infective populations are shown in light blue). If the travel kernel *K* is dominated by distance (*α* small, c.f. S3 Fig), then some of the infectious population in each pixel will relocate to neighboring pixels (white). The result is a higher prevalence in locally sparsely populated pixels. Moreover, a larger local population gradient will allow this phenomenon to persist. Moreover, infection status is recorded by home location, which, under the I-mobility assumption, is equivalent to location when susceptible/recovered. The result is a negative correlation between local population density and attack rate.
(TIF)

S1 Protocol Additional algebraic analyses. Algebraic analyses of: uniform local attack rates for dual mobility assumptions; the relationship between our results and other approximations in the literature [35–37]; convoluted kernel formulations; and calculation of the global transmissibility coefficient.
(PDF)
