# Supplementary material for: Differential mobility and local variation in infection attack rate
Source: PLoS Comput Biol. 2019 Jan 22;15(1):e1006600. doi: 10.1371/journal.pcbi.1006600 (PMC6358099; doi:10.1371/journal.pcbi.1006600)
Supplement: S1 Table — Results for different grid sizes involve aggregation of result obtained at 1km by 1km resolution. In all cases, empty pixels are omitted from calculations. It is therefore possible to obtain a smaller minimum value of attack rate after aggregation. (PDF) [file pcbi.1006600.s001.pdf]

Model				Attack rate				
(R_0, γ, α)	Population	Grid	Mobility ass.	Min.	Max.	Global	CC_{pop}	CC_{grad}
$(1.8, 1/2.6, 0.52)$	Guangzhou	1km by 1km	DM	0.425	0.425	0.425	0	0
			SM	0.3372	0.421	0.4576	0.7469	0.7347
			IM	0.3261	0.9073	0.4077	0.7707	0.8816
		4km by 4km	DM	0.4576	0.4576	0.4576	0	0
			SM	0.5523	0.7259	0.359	0.4214	0.4399
			IM	0.3648	0.5384	0.4076	-0.4651	-0.6883
$(1.8, 1/2.6, 1)$	Guangzhou	1km by 1km	DM	0.425	0.425	0.425	0	0
			SM	0.2909	0.6842	0.4316	-0.5687	-0.149
			IM	0.0355	0.5029	0.3672	0.8213	0.5454
		4km by 4km	DM	0.4576	0.4576	0.4576	0	0
			SM	0.318	0.6657	0.431	0.7668	0.768
			IM	-0.0415	0.5029	0.3687	0.5384	0.4076
$(1.8, 1/2.6, .052)$	Puerto Rico	1km by 1km	DM	0.4251	0.4251	0.4251	0	0
			SM	0.3598	0.4703	0.4231	0.6323	0.738
			IM	0.3405	0.9935	0.4181	-0.7915	-0.8887
		4km by 4km	DM	0.4251	0.4251	0.4251	0	0
			SM	0.3869	0.4591	0.4231	0.8866	0.5259
			IM	0.3709	0.5932	0.4181	0.8193	-0.8887
$(4, 1/10, 0.52)$	Puerto Rico	1km by 1km	DM	0.912	0.912	0.912	0	0
			SM	0.8683	0.9298	0.9111	0.6923	0.7631
			IM	0.8615	1	0.9072	-0.7528	-0.9008
		4km by 4km	DM	0.912	0.912	0.912	0	0
			SM	0.8851	0.9248	0.9111	0.8805	0.4554
			IM	0.8809	0.98	0.9072	0.864	-0.2702

Table 1: Summary statistics for different model parameters, populations and mobility assumptions. Results for different grid sizes involve aggregation of result obtained at 1km by 1km resolution. In all cases, empty pixels are omitted from calculations. It is therefore possible to obtain a smaller minimum value of attack rate after aggregation.
