# Supplementary figures and images for: Differential mobility and local variation in infection attack rate
Source: PLoS Comput Biol. 2019 Jan 22;15(1):e1006600. doi: 10.1371/journal.pcbi.1006600 (PMC6358099; doi:10.1371/journal.pcbi.1006600)

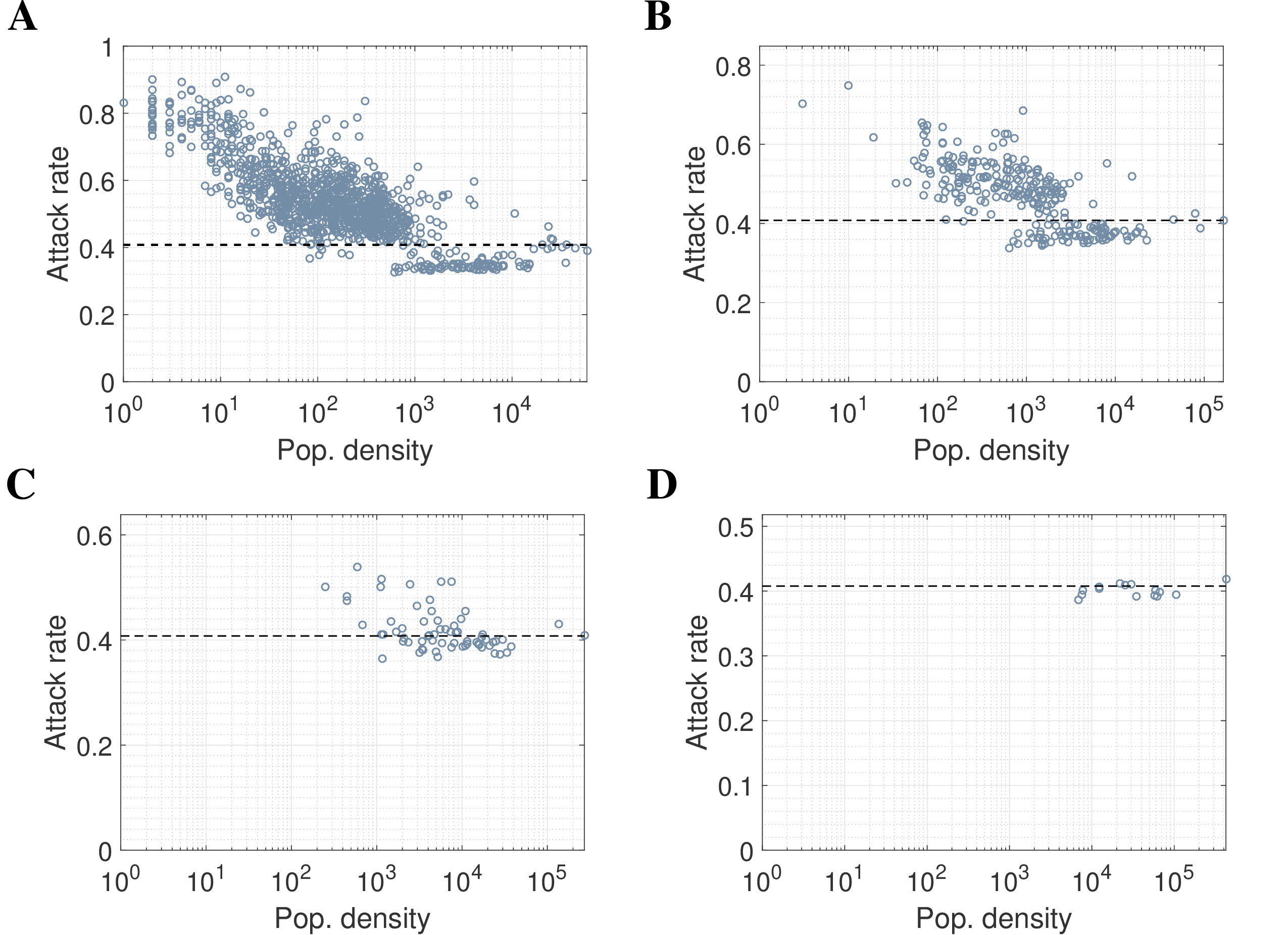

Supplement: S1 Fig — Plots show (A) initial result, aggregated into (B) 2km by 2km, (C) 4km by 4km, and (D) 8km by 8km pixels. (TIFF) [file pcbi.1006600.s002.tiff]

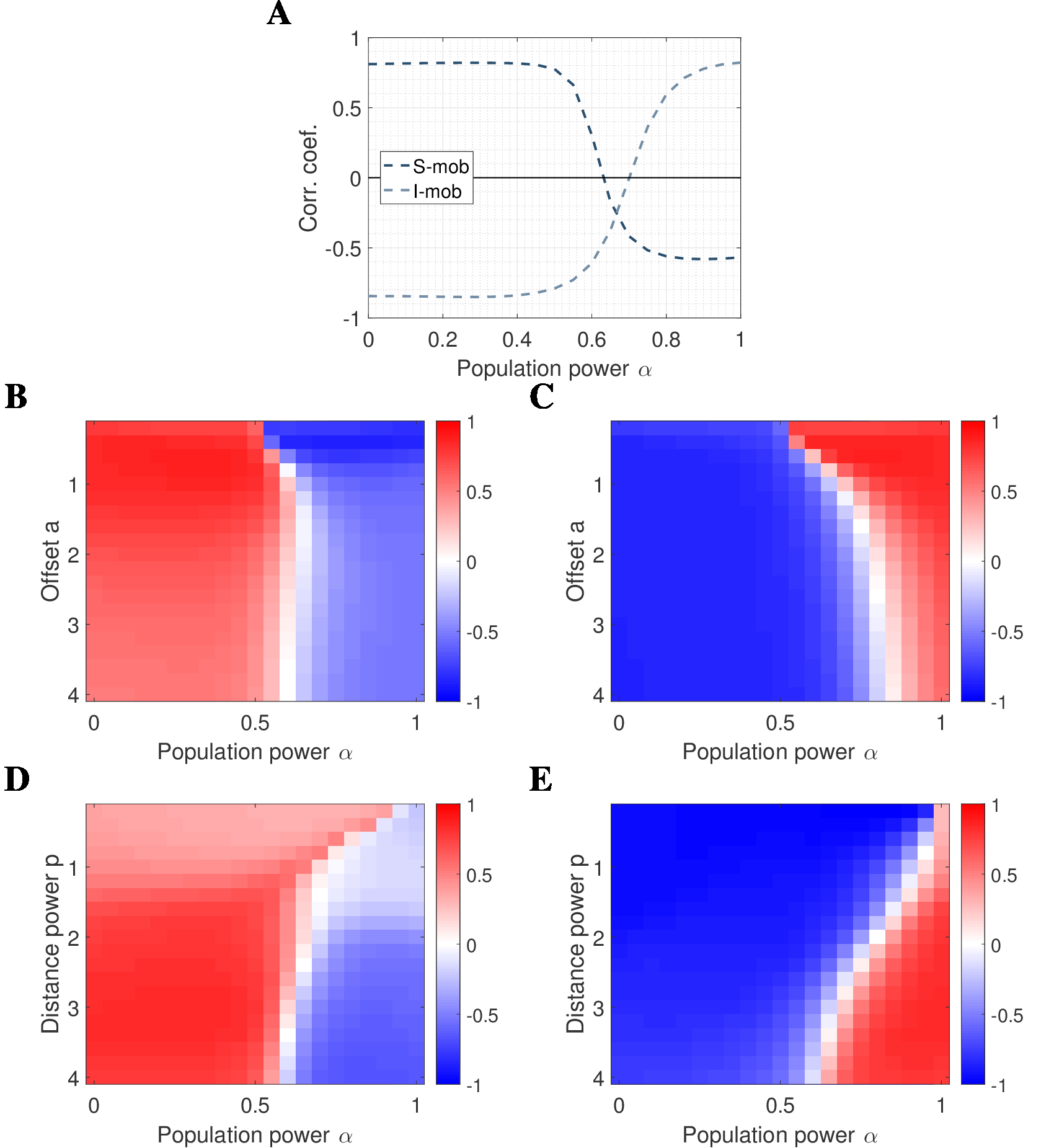

Supplement: S2 Fig — We vary(A) α with a = 0.58 and p = 2.72 fixed, comparing S-mobility with I-mobility (B) a and α, using S-mobility with p = 2.72 fixed, (C) a and α, using I-mobility with p = 2.72 fixed, (D) p and α, using S-mobility with a = 0.58 fixed, and (E) p and α, using I-mobility with a = 0.58 fixed. All fixed parameter values are those used in main result. (TIF) [file pcbi.1006600.s003.tif]

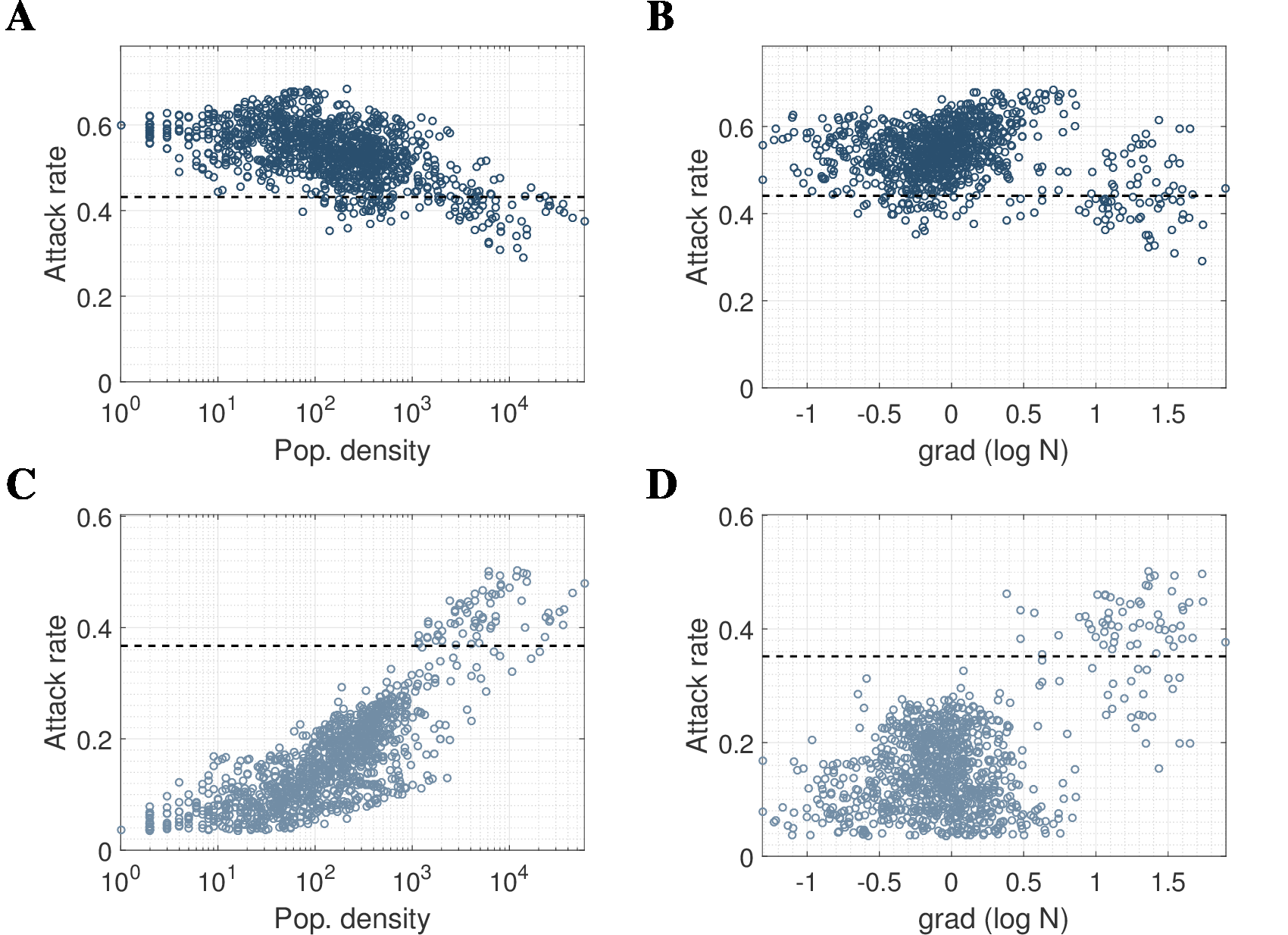

Supplement: S3 Fig — We use (A) S-mobility, with attack rates plotted against population density, (B) S-mobility/gradient, (C) I-mobility/density, and (D) I-mobility/gradient. Other parameters remain as in main result, i.e. a = 0.58, p = 2.72. (TIF) [file pcbi.1006600.s004.tif]

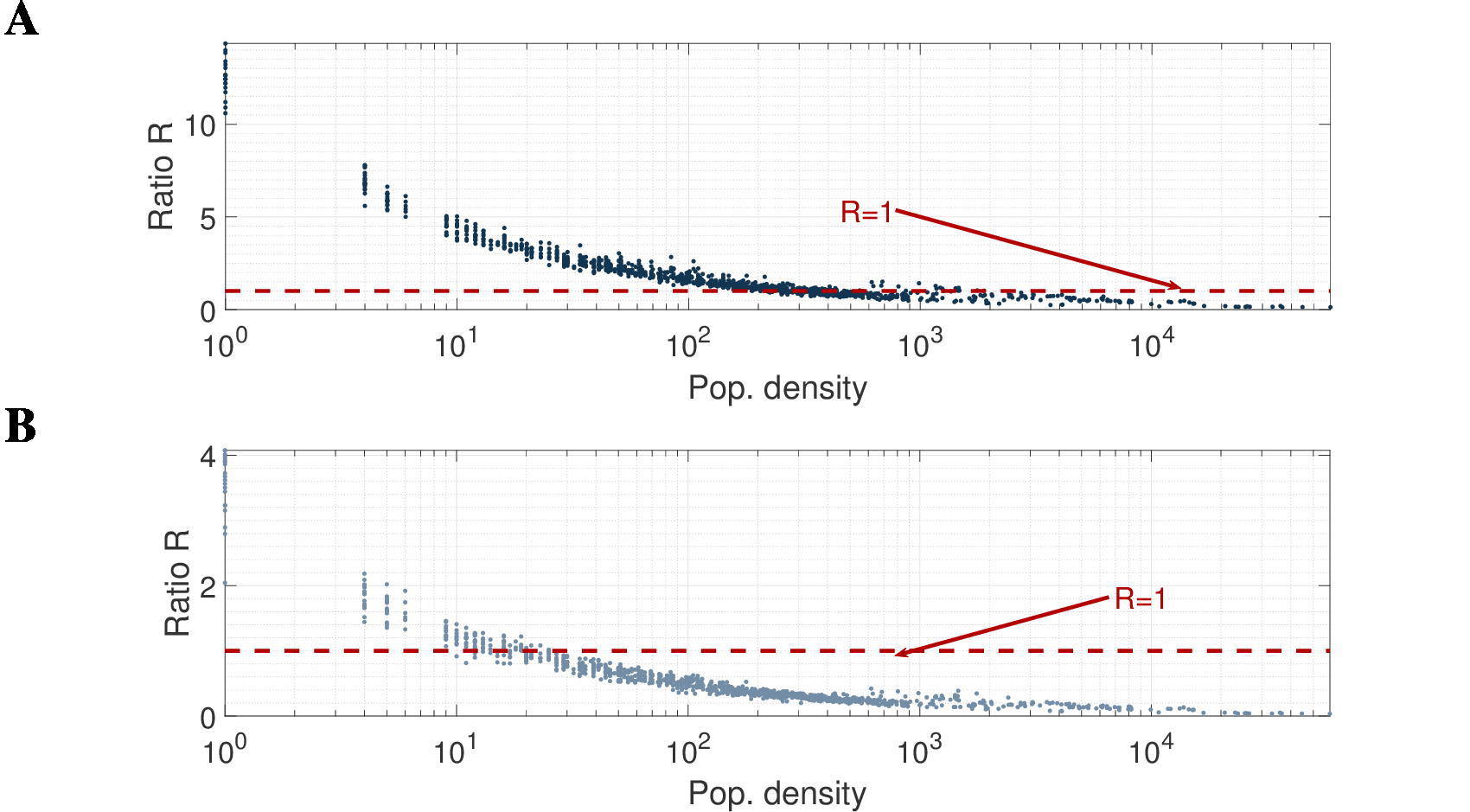

Supplement: S4 Fig — We use (A) S-mobility and (B) I-mobility. All parameters as in main result, i.e. a = 0.58, p = 2.72, α = 0.52. (TIF) [file pcbi.1006600.s005.tif]

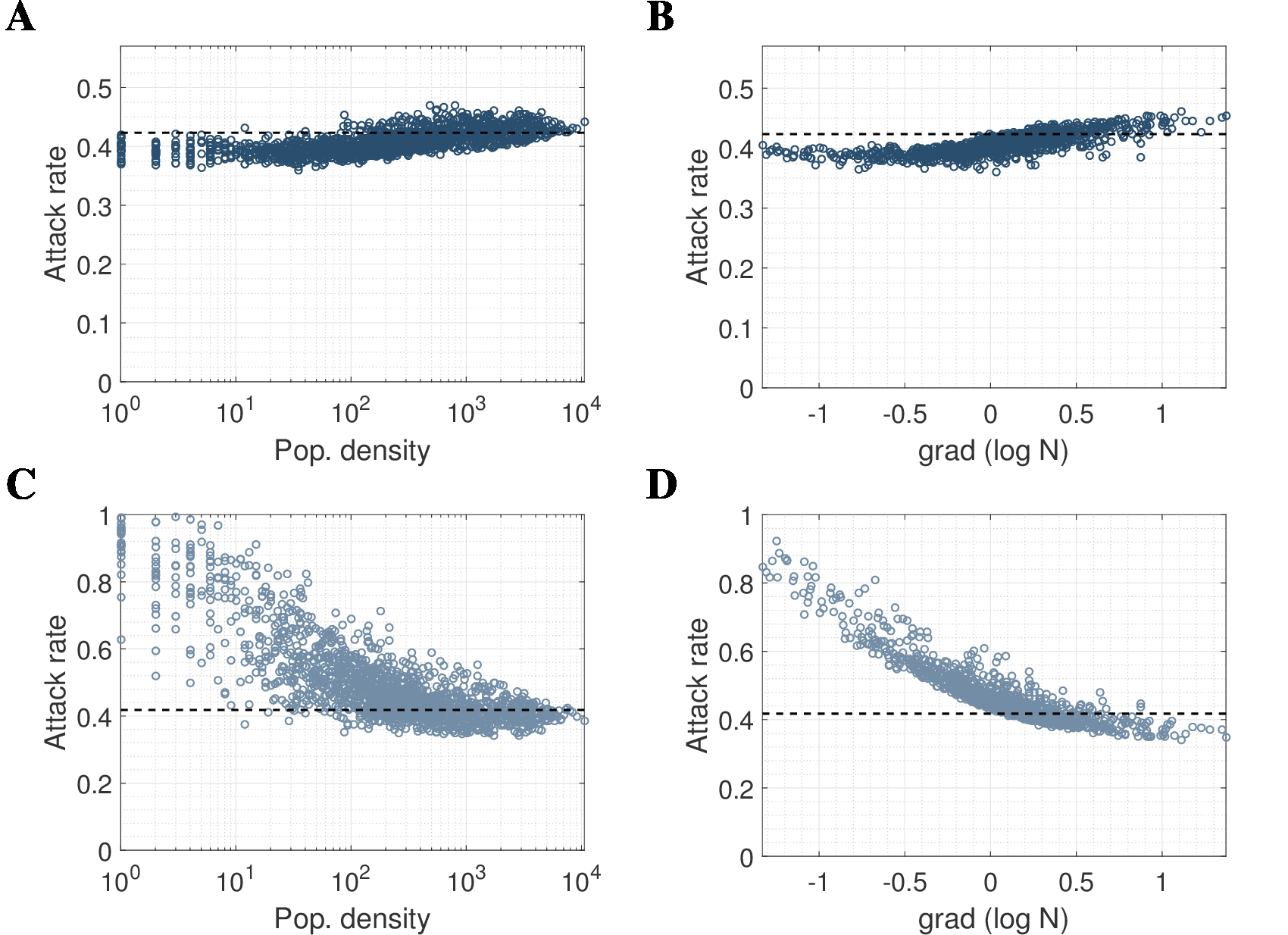

Supplement: S5 Fig — We use a 60km by 60km grid of 1km by 1km pixels, and influenza-like natural history parameters R0 = 1.8, γ = 1/2.6, with (A) S-mobility plotted against population density, (B) S-mobility plotted against log population gradient, (C) I-mobility/density, and (D) I-mobility/gradient. Kernel parameters as in main result, i.e. a = 0.58, p = 2.72, α = 0.52. (TIF) [file pcbi.1006600.s006.tif]

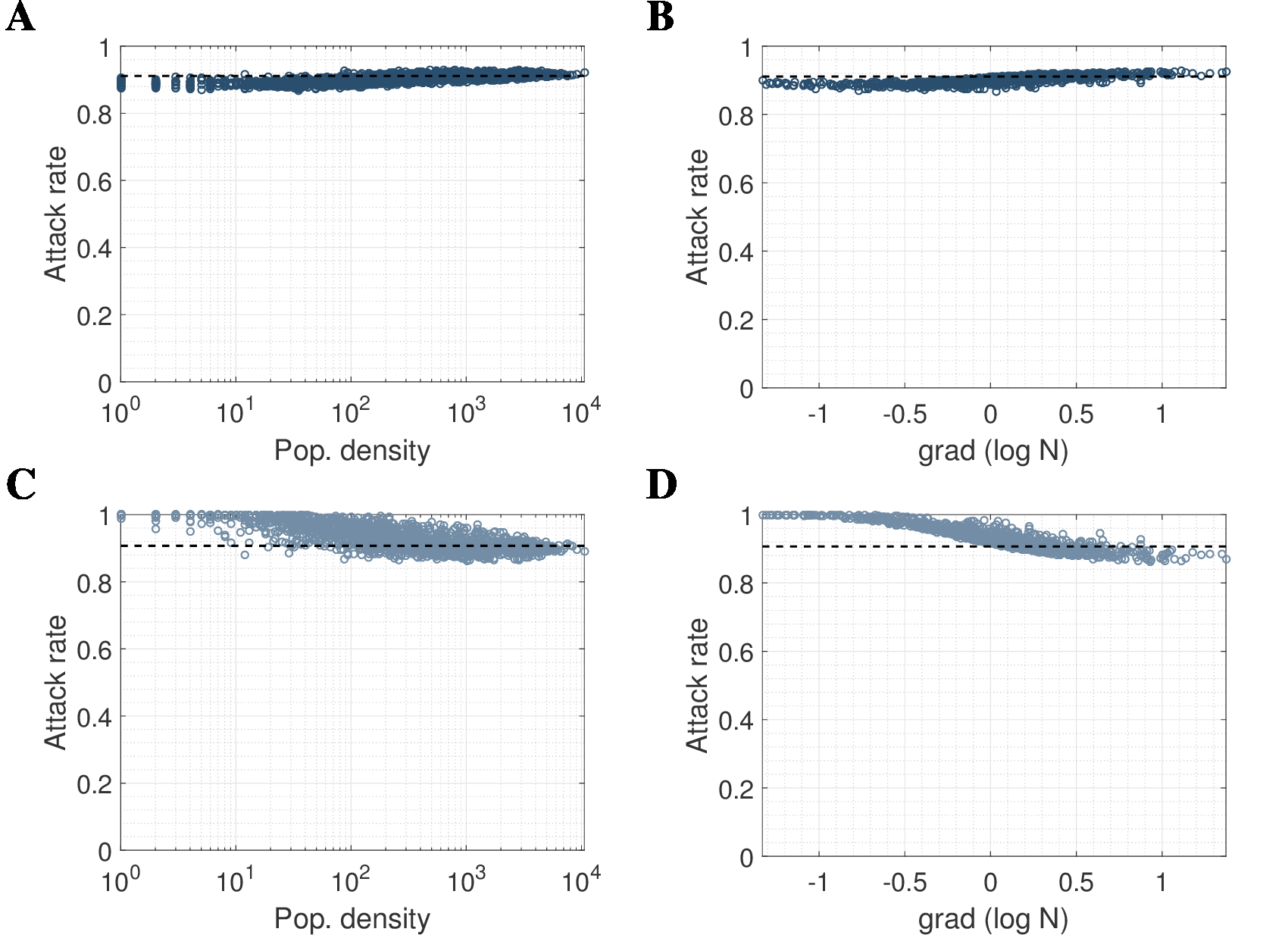

Supplement: S6 Fig — We use a 60km by 60km grid of 1km by 1km pixels, and natural history parameters R0 = 4, γ = 1/10 approximating vector-borne transmission (e.g. Zika, Chikungunya), with (A) S-mobility plotted against population density, (B) S-mobility plotted against log population gradient, (C) I-mobility/density, and (D) I-mobility/gradient. Kernel parameters as in main result, i.e. a = 0.58, p = 2.72, α = 0.52. (TIF) [file pcbi.1006600.s007.tif]

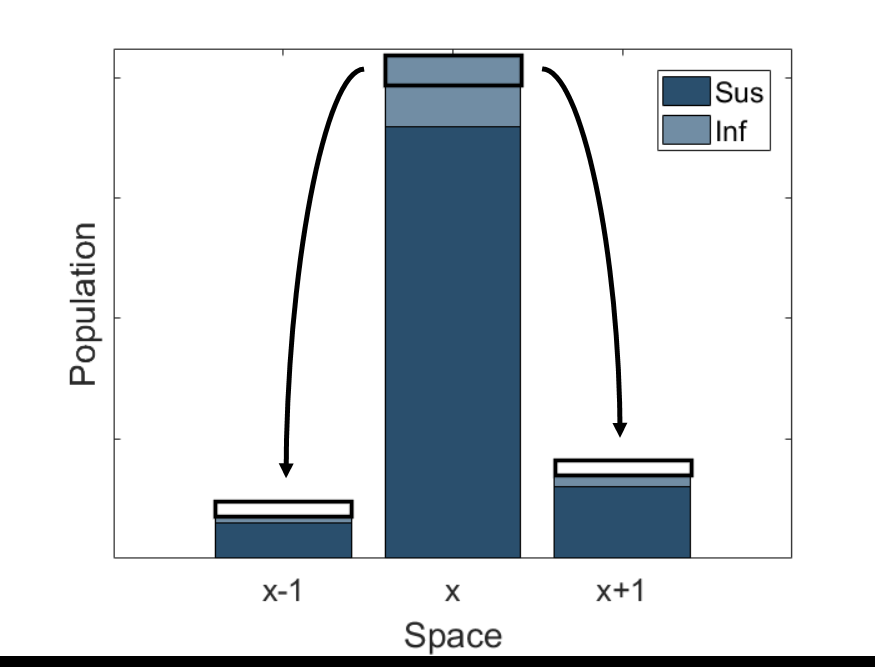

Supplement: S7 Fig — As an example, assume infectious-only mobility and let location x be locally densely populated, with disease prevalence initially proportional to population density (initial infective populations are shown in light blue). If the travel kernel K is dominated by distance (α small, c.f. S3 Fig), then some of the infectious population in each pixel will relocate to neighboring pixels (white). The result is a higher prevalence in locally sparsely populated pixels. Moreover, a larger local population gradient will allow this phenomenon to persist. Moreover, infection status is recorded by home location, which, under the I-mobility assumption, is equivalent to location when susceptible/recovered. The result is a negative correlation between local population density and attack rate. (TIF) [file pcbi.1006600.s008.tif]
