# Supplementary material for: Differential mobility and local variation in infection attack rate
Source: PLoS Comput Biol. 2019 Jan 22;15(1):e1006600. doi: 10.1371/journal.pcbi.1006600 (PMC6358099; doi:10.1371/journal.pcbi.1006600)
Supplement: S1 Protocol — Algebraic analyses of: uniform local attack rates for dual mobility assumptions; the relationship between our results and other approximations in the literature [35–37]; convoluted kernel formulations; and calculation of the global transmissibility coefficient. (PDF) [file pcbi.1006600.s009.pdf]

S1 Protocol: Additional algebraic analyses

Uniform local attack rates for dual mobility assumptions

Citations in this document refer to the References section of the main document.

To show uniformity of attack rate with respect to space, we construct the final size equation for the system. If the final size for age-group a in location i is given by $z_{a,i} = R_{a,i}/N_i$, then

$$\begin{aligned}
\int_0^\infty \frac{\dot{S}_{a,i}}{S_{a,i}} dt &= -\beta \sum_{b,j} L^D_{(a,i)(c,k)} \int_0^\infty \frac{I_{c,k}}{N_k} && (1) \\
\text{where} \quad L_{(a,i)(c,k)} &= K_{ij} \delta_{ab} \frac{\sum_{c,k} K^T_{jk} C_{bc} N_k}{\sum_{d,l} K^T_{jl} N_{(d,l)}} && (2) \\
&= K_{ij} \delta_{ab} \frac{\sum_k K^T_{jk} N_k \sum_c C_{bc}}{\sum_l K^T_{jl} N_l} && (3) \\
\text{so} \quad \log(1 - z_{a,i}) - \log\left(\frac{N_{a,i}}{N_i}\right) &= -\frac{\beta}{\gamma} \sum_{b,j} L^D_{(a,i)(c,k)} z_{c,k} && (4)
\end{aligned}$$

As reasoned in Ref [35] (for the S-mobility FOI with denominator N_i, and in the absence of age-mixing), if there exists a solution $\mathbf{z}$ such that $z_{a,i} = x_a$, i.e. final sizes are independent of space, then we have:

$$\begin{aligned}
\log(1 - z_{a,i}) - \log\left(\frac{N_{a,i}}{N_i}\right) &= -\beta \sum_{b,j} K_{ij} \delta_{ab} \frac{\sum_k K^T_{jk} N_k}{\sum_l K^T_{jl} N_l} \sum_c C_{bc} x_c && (5) \\
&= -\beta \sum_j K_{ij} \sum_c C_{ac} x_c && (6) \\
&= -\beta \sum_c C_{a,c} x_c && (7)
\end{aligned}$$

If the distribution of age-groups is uniform in space, then we have $N_{a,i}/N_i = q_a$, and so, if there exists a solution to the age-only final size equation:

$$\log(1 - \mathbf{x}) - \log \mathbf{q} = -\beta C \mathbf{x} \qquad (8)$$

then $z_{a,i} = x_a$ is a solution to equation (4), and final sizes are uniform in space.

Relationship to other approximations in the literature

It is shown in [35] that susceptible-only mobility induces uniformity of attack rate, using a FOI with normalization by native population. In fact, we can show that uniformity is guaranteed only when all agents are equally mobile, owing to denominator in the force of infection term, which must be corrected to account for spatial mobility within the whole population.

For ease of notation, the following formulae are presented without explicit reference to age-mixing, but this is always included in computational results (c.f. methods for age-explicit formulae). The dual mobility FOI assumes that all agents are fully mobile as described by the kernel K (a stochastic matrix). The dual mobility FOI on an agent resident in pixel i is given by

$$\lambda_i^D = \beta \sum_j K_{ij} \frac{\sum_k K_{jk}^T I_k}{M_j} \quad (9)$$

$$M_j = \sum_l K_{jl}^T N_l \quad (10)$$

where M_j denotes the total population present in pixel j. Crucially, when a model incorporates spatial mobility, we can not say $M_j = N_j$. This FOI assumes frequency-dependent transmission based on constant contacts, and describes the expected dynamics in an agent-based system with explicit travel determined by K.

In the literature, the S- and I-mobility kernels are typically denoted as follows:

$$\lambda_i^S = \beta \sum_j K_{ij} \frac{I_j}{N_j} \quad (11)$$

$$\lambda_i^I = \beta \sum_j K_{ij}^T \frac{I_j}{N_i} \quad (12)$$

We claim that the denominators N_j and N_i do not accurately represent the population present in pixels j and i respectively in high resolution gridded models, owing to spatial mobility. The argument below shows that the classic IM FOI serves as at least as a good approximation when incidence is small, but the SM FOI does not.

Using the above equations, SM and DM both induce uniform cumulative attack rates in space.

The real SM FOI is significantly different to equation (11) in a way that is described by the ratio of total time spent in each pixel and the native population of that pixel.

Consider deriving the S-mobility and I-mobility FOIs from the dual mobility FOI. This involves starting with equation (9) and replacing either the single appearance of K or the single appearance of K^T with the identity matrix (denoted E to avoid confusion with the I_i), and adjusting denominators M_j accordingly:

$$
\begin{aligned}
\lambda_i^S &= \beta \sum_j K_{ij} \frac{\sum_k E_{jk}^T I_k}{I_j + \sum_l K_{jl}^T (N_l - I_l)} && (13) \\
&= \beta \sum_j K_{ij} \frac{I_j}{I_j + \sum_l K_{jl}^T (N_l - I_l)} && (14) \\
&\approx \beta \sum_j K_{ij} \frac{I_j}{\sum_l K_{jl}^T N_l} \quad \text{(first approximation)} && (15) \\
&\approx \beta \sum_j K_{ij} \frac{I_j}{N_j} \quad \text{(second approximation)} && (16) \\
\lambda_i^I &= \beta \sum_j E_{ij} \frac{\sum_k K_{jk}^T I_k}{N_j - I_j + \sum_l K_{jl}^T I_l} && (17) \\
&= \beta \frac{\sum_k K_{ik}^T I_k}{N_i - I_i + \sum_l Kjl^T I_l} && (18) \\
&\approx \beta \sum_j K_{ij}^T \frac{I_j}{N_i} && (19)
\end{aligned}
$$

The denominator in equation (14) is not N_j (the native population of pixel j) but is instead the population *present in pixel* j according to K, when only the non-infectious population is mobile. In the case where this is equal to the native population of pixel j, we have uniformity of attack rates, as seen in the literature (using a similar argument to our proof that the DM FOI induces uniform attack rates).

The denominator in equation (15) is that of the dual mobility assumption. This approximation is valid prevalence is low, i.e. $I_i \ll N_i \ \forall i$, and the absence of dynamic quantities in the denominator yields analytic tractability and faster simulation.

The denominator in equation (16) is simply the native population of that pixel, thus yielding uniform attack rates. This assumption is equivalent to the assumption that the total number of

people leaving each pixel is the same as the total number of people arriving in each pixel, i.e. that the matrix K^T is also stochastic.

This is a weaker assumption but is related to the $N_i \to \infty$ approximation used in [36]. When using the full FOI terms for S- and I-mobility, the only case in which these conventional mobility assumptions induce uniformity of attack rate is when each location is equally visited (in mathematical terms, uniformity of total contact means that the spatial kernel is a stochastic matrix, and the latter requirement is equivalent to the transpose of K also being stochastic, hence K is orthogonal). The notion of normalization by total population present is not new to the literature [37], though is often excluded in the construction of spatial epidemic models.

Convoluted kernel formulations

It is possible to change the mobility assumption in an existing model via an effective or convoluted kernel L such that replacing K with L in a given explicit FOI is equivalent to a change of mobility assumption. In fact, we can write any spatially explicit FOI in the form:

$$\lambda_i = \beta \sum_j L_{ik} \frac{I_k}{N_k}$$

for some matrix L. This formulation is essential in final size calculations. Then, for example, the convoluted D-mobility kernel L^D is given by $L^D_{ik} = \sum_j K_{ij} K^T_{jk} N_j / M_j$, where $M_j = \sum_l K_{jl} N_l$, as in the main text. When using low-prevalence approximations, this can be done prior to numerical simulation, and so requires minimal additional modification to existing model codes. Note that this representation of FOI is structurally equivalent to the S-mobility second approximation given in equation (16), though the effective travel kernel L is now non-isotropic.

Global transmissibility coefficient

In all simulations, we use the next generation matrix (NGM) method to derive a global transmissibility parameter β that yields our desired global R_0. NGMs are derived from λ_i, evaluated at disease-free equilibrium (DFE). We can show that, in all 3 cases, using the approximations to S- and I-mobility given in equations (15) and (19) the value of β obtained is

equal to that of the spatially heterogeneous system, maintaining heterogeneity in age only.

In the I-mobility case, the NGM is given by

$$G^I_{(a,i)(b,j)} = \frac{\beta}{\gamma}\frac{N_{(a,i)}}{N_i}C_{ab}K^T_{ij} \tag{20}$$

Since K is a stochastic matrix, we have $\lambda_1(K) = 1$ and so $\lambda_1(K^T) = 1$, thus the dominant eigenvalue of G^I is equal to the dominant eigenvalue of

$$G^{age}_{ab} = \frac{\beta}{\gamma}\frac{N_a}{N_{total}}C_{ab} \tag{21}$$

Using S-mobility, we have

$$\begin{aligned}
G^S_{(a,i)(c,k)} &= \frac{\beta}{\gamma}N_{a,i}C_{ac}K_{ik}\frac{1}{\sum_{d,l}K^T_{kl}N_{(d,l)}} && (22)\\
&= \frac{\beta}{\gamma}C_{ac}\frac{K^T_{ki}N_{a,i}}{\sum_{d,l}K^T_{kl}N_{(d,l)}} && (23)\\
&= \frac{\beta}{\gamma}C_{ac}X_{(a,i),(c,k)} && (24)\\
\text{where} \quad X_{(a,i)(c,k)} :&= \frac{K^T_{ki}N_{a,i}}{\sum_{d,l}K^T_{kl}N_{(d,l)}} && (25)
\end{aligned}$$

Note that X is a stochastic matrix, and so the dominant eigenvalue of G^S is equal to the dominant eigenvalue of G^{age}.

A similar argument applies to dual mobility, where we have

$$\begin{aligned}
G^D_{(a,i)(c,k)} &= \frac{\beta}{\gamma}N_{a,i}C_{ac}\sum_j K_{ij}\frac{K^T_{jk}}{\sum_{d,l}K^T_{kl}N_{(d,l)}} && (26)\\
&= \frac{\beta}{\gamma}C_{ac}\sum_j K_{kj}\frac{K^T_{ji}N_{a,i}}{\sum_{d,l}K^T_{kl}N_{(d,l)}} && (27)\\
&= \frac{\beta}{\gamma}C_{ac}Y_{(a,i),(c,k)} && (28)\\
\text{where} \quad Y_{(a,i)(c,k)} :&= \sum_j K_{kj}\frac{K^T_{ji}N_{a,i}}{\sum_{d,l}K^T_{kl}N_{(d,l)}} && (29)
\end{aligned}$$

Here, since Y is the product of 2 stochastic matrices, it must itself be stochastic, and so the

dominant eigenvalue of G^D is also equal to the dominant eigenvalue of G^{age}.

The arguments presented above for susceptible-only and dual mobility require that the same travel kernel K be used to describe the movement of all age groups, i.e. K_{ij} be independent of a, b, c, d. It can be verified computationally that age-dependent mobility can indeed induce different values of β to the spatially heterogeneous model, in all cases other than pure infectious-only mobility. We reserve a detailed analysis of this scenario for a subsequent study.
